# Plant-based dietary index and body weight in people with type 1 diabetes: a secondary analysis of a randomized clinical trial

**DOI:** 10.3389/fnut.2025.1605769

**Published:** 2025-05-22

**Authors:** Hana Kahleova, Ilana Fischer, Reagan Smith, Joseph Himmelfarb, Tatiana Znayenko-Miller, Richard Holubkov, Neal D. Barnard

**Affiliations:** ^1^Physicians Committee for Responsible Medicine, Washington, DC, United States; ^2^School of Medicine, University of Utah, Salt Lake City, UT, United States; ^3^George Washington University School of Medicine and Health Sciences, Washington, DC, United States

**Keywords:** diet quality, nutrition, plant-based, portion-controlled, vegan, Plant-based dietary index, Body weight, type 1 diabetes

## Abstract

**Objective:**

This secondary analysis tested the relationship of a plant-based diet index (PDI), healthful PDI (hPDI), and unhealthful PDI (uPDI), with weight loss in adults with type 1 diabetes.

**Methods:**

Fifty-eight adults with type 1 diabetes were randomized to follow an *ad libitum* low-fat vegan (*n* = 29) or a portion-controlled, energy-restricted diet (*n* = 29) for 12 weeks. Food records were analyzed and PDI indices were calculated. A repeated measure ANOVA, Spearman correlations, and a linear regression model were used for statistical analysis.

**Results:**

The PDI score increased on the vegan diet (*p* < 0.001) from 51.8 to 60.4, and did not change on the portion-controlled diet [effect size +6.0 (95% CI + 1.0 to +10.9); *p* = 0.02]; the hPDI increased on both diets, more on the vegan diet [effect size +9.1 (95% CI + 3.7 to +14.5); *p* = 0.002]; and uPDI increased on the vegan diet, and did not change on the portion-controlled diet [effect size +7.3 (95% CI + 1.9 to +12.7); *p* = 0.01]. Changes in PDI and hPDI scores correlated with changes in body weight [*r* = −0.35; *p* = 0.04 for PDI; and *r* = −0.52; *p* = 0.001 for hPDI], even after adjustment for changes in energy intake [*r* = −0.37; *p* = 0.04 for PDI; and *r* = −0.53; *p* = 0.001 for hPDI]. An increase in hPDI by 6.1 points was associated with a 1-kg weight loss (*p* = 0.01). There was no association between the changes in uPDI and changes in body weight (*r* = −0.07; *p* = 0.68).

**Conclusion:**

The study results suggest that replacing animal foods with plant foods is an effective strategy for weight loss in adults with type 1 diabetes. The inclusion of “unhealthy” plant-based foods did not impair weight loss, and these benefits were independent of energy intake.

**Clinical trial registration:**

ClinicalTrials.gov, identifier NCT04944316.

## Introduction

People following plant-based diets have been shown to have lower body weight and lower risk of type 2 diabetes. Based on observational data, an attempt has been made to categorize the healthfulness of plant foods, using plant-based (PDI), unhealthful (uPDI), and healthful (hPDI), dietary indices (1). However, a previous randomized trial in overweight adults has shown that eating plant foods (both from the so-called healthful and unhealthful categories), instead of animal products, was associated with weight loss (2). The potential role, if any, of PDI in adults with type 1 diabetes has yet to be explored.

A previously published randomized clinical trial showed that, compared to a portion-controlled diet, a vegan diet resulted in a clinically significant weight loss and improvements in insulin sensitivity in adults with type 1 diabetes (3). This secondary analysis tested the associations of PDI, uPDI, and hPDI with changes in body weight in adults living with type 1 diabetes.

## Methods

The study methods have been described in detail previously (3). Briefly, this randomized trial took place in 2021–2022 in Washington, DC. Adults diagnosed with type 1 diabetes were enrolled. The study protocol was approved by the Chesapeake Institutional Review Board on February 03, 2021. The study participants signed an informed consent.

### Dietary interventions

The participants were randomized in a 1:1 ratio to an *ad libitum* low-fat vegan diet (*n* = 29) or a portion-controlled, energy-restricted diet (*n* = 29). The low-fat vegan diet was free of all animal products and consisted of only plant foods. The portion-controlled diet emphasized portion control and keeping carbohydrate intake steady. Both groups met weekly online and were supported by registered dietitians. The participants tracked all their meals via Cronometer (Cronometer Inc., Revelstoke, Canada). The participants were instructed to keep their physical activity and their medications constant throughout the study. Insulin was modified in response to repeated hypoglycemia. All outcomes were measured at week 0 and 12.

At week 0 and 12, a detailed food record was filled out for three consecutive days and analyzed by a registered dietitian certified in the Nutrition Data System for Research (4). The PDI, uPDI, and hPDI were assessed (1). “Healthful” plant-based foods, as defined by the PDI system, include fruits, vegetables, whole grains, nuts, legumes, oils, coffee and tea, and “unhealthful” plant-based foods include fruit juice, sugar-sweetened beverages, refined grains, potatoes, and sweets (1).

### Statistical analysis

A repeated-measure ANOVA model was performed by a statistician blinded to the dietary interventions. Paired comparison t-tests were used to assess any within-group changes. Spearman correlations were used to test the relationship between changes in PDI, hPDI, and uPDI and in body weight, first unadjusted, and then adjusted for changes in energy intake, and also correlations between changes in PDI, hPDI, and uPDI, and changes in energy intake. Bonferroni correction was used, and *p*-values less than 0.006 (0.05/9) were presented as significant. A linear regression model was used to determine whether changes in PDI and hPDI were independent predictors of weight loss.

## Results

### Characteristics of the participants

Fifty-eight adults were randomized to follow a vegan (*n* = 29) or a portion-controlled (*n* = 29) diet (Supplementary Figure 1). The attrition rates were similar in both groups. There was no difference between the groups in energy intake.

### PDI, hPDI, uPDI

The PDI score increased on the vegan diet (*p* < 0.001) from 51.8 to 60.4, and did not change on the portion-controlled diet [effect size +6.0 (95% CI + 1.0 to +10.9); *p* = 0.02]; the hPDI increased on both diets, more on the vegan diet [effect size +9.1 (95% CI + 3.7 to +14.5); *p* = 0.002]; and uPDI increased on the vegan diet, and did not change on the portion-controlled diet [effect size +7.3 (95% CI + 1.9 to +12.7); *p* = 0.01]. As expected, compared to the portion-controlled diet, the intake of all animal foods was significantly reduced on the vegan diet. Furthermore, the scores for the “healthful” plant foods changed as follows: legumes, whole grains, and fruits significantly increased, while the scores for vegetable oils and nuts significantly decreased (as the consumption of these foods decreased) on the vegan diet. On the portion-controlled diet, only the score for whole grains increased. The scores for “unhealthful” plant foods had no significant change on either diet, with the exception of a reduced score in refined grains on the portion-controlled diet (see Table 1).

**Table 1:** Plant-based dietary index (PDI), healthful (hPDI), and unhealthful plant-based dietary index (uPDI), and the individual food components at baseline and 12 weeks.

Variable	Vegan group			Portion-Control group			Effect size	*P*-value
	Week 0	Week 12	Change	Week 0	Week 12	Change		
PDI	51.8 (49.0–54.6)	60.4 (58.5–62.4)	+8.7 (+5.2 to +12.1)***	54.3 (50.7–57.9)	57.0 (53.0–61.1)	+2.7 (−1.1 to +6.6)	+6.0 (+1.0 to +10.9)	0.02
hPDI	54.1 (50.2–58.0)	67.1 (64.3–69.9)	+13 (+8.5 to +17.5)***	58.7 (54.7–62.6)	62.5 (57.8–67.3)	+3.9 (+0.7 to +7.0)*	+9.1 (+3.7 to +14.5)	0.002
uPDI	54.6 (51.3–57.8)	60.9 (58.1–63.7)	+6.3 (+2.8 to +9.9)**	54.1 (49.8–58.3)	53.1 (50.1–56.1)	−0.9 (−5.4 to +3.5)	+7.3 (+1.9 to +12.7)	0.01
Food components for PDI (points)								
Fruits	2.9 (2.2–3.7)	3.9 (3.3–4.5)	+0.9 (+0.3 to +1.6)**	3.1 (2.3–3.8)	3.2 (2.6–3.9)	+0.2 (−0.7 to +1.1)	+0.8 (−0.3 to +1.8)	0.14
Vegetables	3.1 (2.3–3.8)	3.8 (3.1–4.5)	+0.7 (−0.1 to +1.5)	2.9 (2.2–3.7)	3.3 (2.6–4.0)	+0.4 (−0.6 to +1.3)	+0.4 (−0.8 to +1.5)	0.53
Whole grains	3.1 (2.3–3.9)	4.2 (3.6–4.9)	+1.1 (+0.02 to +2.2)*	2.9 (2.2–3.6)	3.8 (3.2–4.4)	+0.9 (+0.1 to +1.7)*	+0.2 (−1.1 to +1.6)	0.73
Nuts	2.1 (1.4–2.9)	1.0 (1.0–1.0)	−1.1 (−1.9 to −0.4)**	3.4 (2.6–4.2)	3.6 (3.0–4.2)	+0.2 (−0.8 to +1.1)	−1.3 (−2.4 to −0.1)	0.03
Legumes	2.0 (1.2–2.8)	4.7 (4.3–5.0)	+2.7 (+1.9 to +3.4)***	3.3 (2.5–4.1)	3.8 (3.2–4.5)	+0.5 (−0.5 to +1.6)	+2.1 (+0.9 to +3.4)	0.001
Vegetable oils	3.3 (2.8–3.9)	1.0 (1.0–1.0)	−2.3 (−2.9 to −1.8)***	2.7 (1.8–3.5)	3.1 (2.2–3.9)	+0.4 (−0.7 to +1.5)	−2.7 (−3.9 to −1.6)	<0.001
Coffee and tea	3.1 (2.3–3.8)	2.2 (1.6–2.9)	−0.8 (−1.7 to +0.01)	2.9 (2.1–3.7)	2.2 (1.5–2.8)	−0.7 (−1.5 to +0.1)	−0.1 (−1.2 to +1.0)	0.82
Fruit juice	2.8 (2.0–3.5)	2.4 (1.7–3.2)	−0.3 (−1.5 to +0.8)	2.8 (1.9–3.7)	2.7 (1.8–3.5)	−0.2 (−1.2 to +0.9)	−0.2 (−1.7 to +1.3)	0.83
Sugar sweetened beverages	2.1 (1.3–2.9)	1.6 (0.9–2.3)	−0.5 (−1.3 to +0.3)	1.8 (1.0–2.6)	1.4 (0.8–2.0)	−0.4 (−1.5 to +0.7)	−0.1 (−1.4 to +1.2)	0.89
Refined grains	2.9 (2.2–3.6)	2.8 (2.1–3.6)	−0.1 (−1.0 to +0.9)	3.1 (2.3–3.9)	2.1 (1.4–2.8)	−1.0 (−1.9 to −0.2)*	+0.9 (−0.3 to +2.2)	0.13
Potatoes	3.3 (2.4–4.1)	2.8 (1.8–3.7)	−0.5 (−1.9 to +0.9)	1.8 (1.1–2.5)	2.5 (1.6–3.4)	+0.7 (−0.5 to +1.8)	−1.1 (−2.9 to +0.6)	0.19
Sweets	2.8 (2.0–3.6)	2.0 (1.3–2.7)	−0.8 (−1.8 to +0.2)	3.2 (2.6–3.9)	3.6 (3.0–4.2)	+0.4 (−0.5 to +1.2)	−1.1 (−2.4 to +0.2)	0.09
Animal fats	3.2 (2.3–4.0)	5.0 (5.0–5.0)	+1.8 (+1.0 to +2.7)***	3.5 (2.6–4.4)	3.4 (2.6–4.1)	−0.1 (−1.0 to +0.7)	+2.0 (+0.8 to +3.1)	0.001
Dairy	2.7 (2.0–3.4)	5.0 (5.0–5.0)	+2.3 (+1.6 to +3.0)***	3.3 (2.5–4.1)	3.3 (2.4–4.2)	0.0 (−0.8 to 0.8)	+2.3 (+1.3 to +3.2)	<0.001
Eggs	3.1 (2.4–3.8)	5.0 (5.0–5.0)	+1.9 (+1.2 to +2.6)***	3.3 (2.4–4.2)	4.4 (3.8–4.9)	+1.1 (−0.02 to +2.1)	+0.8 (−0.4 to +2.1)	0.18
Meat	2.6 (1.8–3.3)	5.0 (5.0–5.0)	+2.4 (+1.7 to +3.2)***	3.7 (3.0–4.4)	3.4 (2.5–4.3)	−0.4 (−0.9 to +0.2)	+2.8 (+1.9 to +3.7)	<0.001
Seafood	3.8 (2.9–4.7)	5.0 (5.0–5.0)	+1.2 (+0.3 to +2.1)*	3.6 (2.7–4.5)	4.5 (3.9–5.1)	+0.9 (−0.02 to +1.8)	+0.3 (−0.9 to +1.6)	0.58

Data are presented as means with 95% confidence intervals. *p < 0.05; **p < 0.01; ***p < 0.001.

### Associations with changes in body weight

The participants lost 5.2 kg on the vegan diet (*p* < 0.001), while there was no change on the portion-controlled diet [effect size −4.3 kg (−6.1 to −2.4); *p* < 0.001]. The changes in PDI and hPDI scores were associated with changes in body weight (*r* = −0.35; *p* = 0.04 for PDI; and r = −0.52; *p* = 0.001 for hPDI) and remained largely unchanged after adjustment for changes in energy intake (*r* = −0.37; *p* = 0.04 for PDI; and *r* = −0.53; *p* = 0.001 for hPDI]. There was no correlation between the changes in uPDI and changes in body weight [*r* = −0.07; *p* = 0.68; see Table 2). A 1-kg weight loss was associated with an increase in hPDI by 6.1 points (*p* = 0.01). No correlation was observed between changes in PDI, hPDI, or uPDI, and changes in energy intake (Table 2).

**Table 2:** Spearman correlations between changes in body weight and changes in the plant-based dietary index (PDI), healthy plant-based dietary index (hPDI), and unhealthy plant-based dietary index (uPDI).

Variable	Δ Body weight	Δ Body weight, adj. For Δ Energy intake	Δ Energy intake
PDI	*r* = −0.35; *p* = 0.04	*r* = −0.37; *p* = 0.03	*r* = +0.15; *p* = 0.40
hPDI	*r* = −0.52; *p* = 0.001	*r* = −0.53; *p* = 0.001	*r* = +0.07; p = 0.68
uPDI	*r* = −0.07; *p* = 0.68	*r* = −0.08; *p* = 0.67	*r* = +0.05; *p* = 0.75

After Bonferroni correction, *p*-values less than 0.006 (0.05/9) were considered significant.

## Discussion

The study demonstrated that replacing animal products with plant foods resulted in weight loss in adults with type 1 diabetes. An increase in hPDI was a predictor of weight loss, independent of changes in energy intake. No association was observed between changes in PDI, hPDI, or uPDI, and changes in energy intake, suggesting that the health benefits of replacing animal products with plant foods are independent of energy intake. These findings are consistent with previous studies.

In a large observational study, higher PDI and hPDI scores correlated with a reduced risk of type 2 diabetes; however, no correlation was observed for uPDI (5). Similar results came from a metabolomic analysis (6). This is, of course, not surprising, because the PDI was developed by identifying dietary factors associated with diabetes in these same cohorts. A previous randomized clinical trial in overweight adults showed, however, that the increase in all three plant-based indices correlated with weight loss. Instead of consuming animal foods, all plant foods (both “healthful” and “unhealthful”) resulted in weight loss (2). While oil and nuts are classified among the “healthful” plant foods, their consumption significantly decreased on the low-fat vegan diet, which likely contributed to the observed weight loss. There was no significant change in the consumption of the individual “unhealthful” plant food components on the vegan diet, which means that the increase in uPDI was driven by excluding animal products, so the absence of an association between changes in uPDI and changes in body weight in this study is not surprising.

The study has some important strengths, mainly the randomized, parallel design. The limitations include the collection of self-reported food records, cumbersome meal and blood glucose monitoring, which contributed to a substantial dropout rate.

In conclusion, these findings suggest that instead of consuming animal foods, eating plant foods, both “healthful” and “unhealthful,” may be an effective strategy for weight loss in adults with type 1 diabetes. The inclusion of “unhealthful” plant foods did not impair weight loss, and these benefits were independent of energy intake.

## Data Availability

The raw data supporting the conclusions of this article will be made available by the authors, without undue reservation.
