# Supplementary figures and images for: Plant-based dietary index and body weight in people with type 1 diabetes: a secondary analysis of a randomized clinical trial
Source: Front Nutr. 2025 May 22;12:1605769. doi: 10.3389/fnut.2025.1605769 (PMC12137060; doi:10.3389/fnut.2025.1605769)

Suppl. Fig. 1 Participant Flow Chart through the Trial.

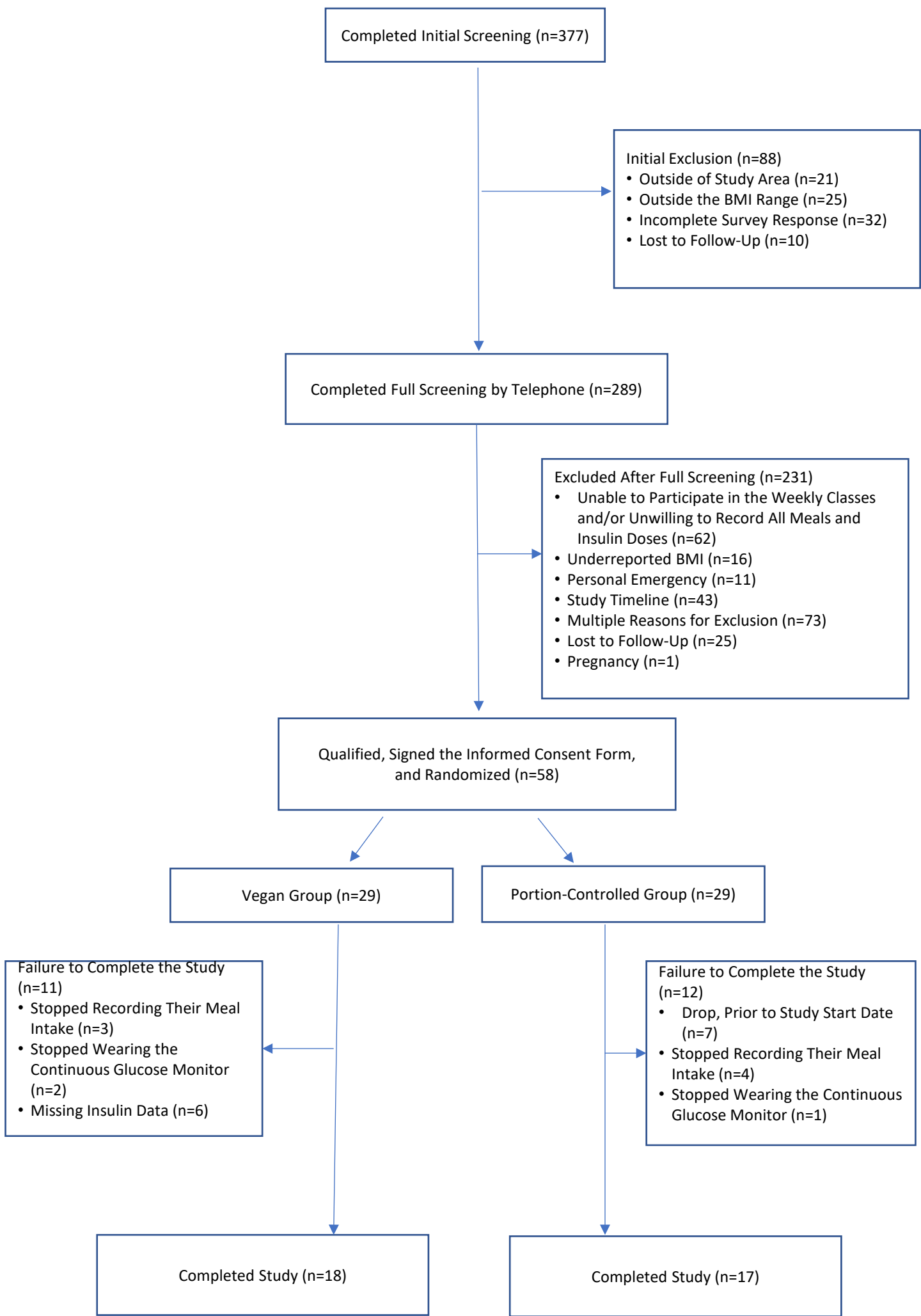

Supplement: Supplementary file 1 [file Image_1.pdf]
